# Genetic relationship between rheumatoid arthritis and cardiovascular diseases: A systematic review of Mendelian randomization studies

**DOI:** 10.1007/s00508-024-02392-8

**Published:** 2024-07-26

**Authors:** Mathias Ausserwinkler, Sophie Gensluckner, Andreas Voelkerer, Jens Thiel, Hans-Jörg Neumann, Maria Flamm, Christian Datz, Elmar Aigner, Bernhard Wernly

**Affiliations:** 1 Department of Internal Medicine, Elisabethinen Hospital Klagenfurt, Klagenfurt, Austria; 2 First Department of Medicine, University Clinic Salzburg, Paracelsus Medical University, Salzburg, Austria; 3 Department of Internal Medicine, General Hospital Oberndorf, Teaching Hospital of the Paracelsus Medical University Salzburg, Salzburg, Austria; 4 Division of Rheumatology and Clinical Immunology, Department of Internal Medicine, Medical University Graz, Graz, Austria; 5 Clinic for Rheumatology and Clinical Immunology, University Hospital Freiburg, Faculty of Medicine, Freiburg, Germany; 6 Institute of General Practice, Family Medicine and Preventive Medicine, Paracelsus Medical University, Strubergasse 21, 5020 Salzburg, Austria

**Keywords:** Rheumatoid arthritis, Inflammatory joint disease, Cardiovascular diseases, Mendelian randomization, Genetic causality, Chronic inflammation

## Abstract

**Objective:**

Rheumatoid arthritis (RA) is recognized as a chronic autoimmune disorder with systemic inflammation and joint damage. Its potential role as a risk factor for cardiovascular diseases (CVD) is increasingly noted. This review delves into the causal relationship between RA and CVD, with Mendelian randomization (MR) offering a genetic perspective.

**Methods:**

An extensive search was conducted in PubMed, Cochrane and Web of Science to identify MR studies addressing the RA-CVD link. Out of 530 studies, 9 met the inclusion criteria, which were rigorously assessed using a critical appraisal checklist. These were further stratified by a sensitivity analysis into categories reflecting the strength of their evidence, from not evaluable to robust.

**Results:**

From the nine included studies, eight supported a causal association between RA and an increased risk of CVD, specifically coronary artery disease (CAD) and one did not support a link between RA and heart failure. The results suggest that genetic factors associated with RA may contribute to an elevated risk for CVD. Chronic inflammation, prevalent in RA, emerges as a key mediator in this connection.

**Conclusion:**

The systematic review corroborates a genetic causal link between RA and CVD, as evidenced by eight of the nine MR studies reviewed. This suggests a need for integrated cardiovascular risk management in the treatment of RA patients. The findings advocate considering anti-inflammatory treatment that can reduce cardiovascular risk. The overarching evidence signifies a potential direction for new therapeutic strategies aimed at enhancing cardiovascular health in RA patients.

**Supplementary Information:**

The online version of this article (10.1007/s00508-024-02392-8) contains supplementary material, which is available to authorized users.

## Key messages

1. Genetic factors in rheumatoid arthritis (RA) elevate cardiovascular risk, indicating a causal relationship.
2. Integrated cardiovascular care is crucial for managing RA patients.
3. Using anti-inflammatory treatment may help reduce cardiovascular risk in RA patients.

## Introduction

RA is a chronic autoimmune disease characterized by systemic inflammation and synovial joint destruction. It affects approximately 1% of the global population, with a higher prevalence among women [1]. While RA primarily targets the joints, accumulating evidence suggests that it also exerts systemic effects, impacting various organ systems beyond the musculoskeletal system [2]. Of particular concern is the well-established association between RA and cardiovascular diseases (CVD), which has garnered increasing attention in recent years. Individuals with RA face a significantly elevated risk of developing CVD, including conditions such as atherosclerosis, coronary artery disease, myocardial infarction, stroke and heart failure [3]. This is also increasingly being demonstrated for other autoimmune diseases [4]. Multiple factors contribute to this heightened cardiovascular risk among RA patients, including chronic inflammation, traditional cardiovascular risk factors and the potential side effects of RA medication, especially glucocorticoids [5]. Chronic inflammation, a hallmark of RA, is increasingly recognized as a key driver in the development and progression of atherosclerosis, making RA a unique model for exploring the complex interplay between chronic inflammatory diseases and CVD [5]. There are data for RA showing that achieving a state of low disease activity or remission leads to a cardiovascular risk comparable to that of the general population [6].

Specific SNPs such as rs2476601 (PTPN22), rs1801274 (FCGR2A), rs651007 (IL6R) and rs10774624 (TNFAIP3) are associated with genes regulating inflammation and immune responses, highlighting their role in the genetic mechanisms underlying RA and the cardiovascular implications [7–10]. This overview underlines the importance of understanding genetic variations to elucidate disease pathways and potential therapeutic targets [8–10]. To investigate the causal relationship between RA and CVD and to decipher potential genetic mechanisms, researchers have turned to Mendelian randomization (MR) studies. This is a powerful and innovative approach that leverages genetic variants as instrumental variables to estimate causal associations between exposure and outcomes [11, 12]. Unlike traditional observational studies, MR studies are less susceptible to confounding and reverse causality, offering a robust method for assessing causality in complex disease relationships. These genetic instruments help to understand whether RA causally contributes to the development of CVD or if the observed association is confounded by shared risk factors. By employing MR connections between RA-related inflammation, genetic predisposition and the collective impact on CVD are investigated [12].

Advancements in genetics and the availability of large-scale genome-wide association studies (GWAS) datasets have facilitated the application of MR to explore the causal links between RA and CVD. These studies aim to explain whether the systemic inflammation and genetic factors associated with RA play a direct role in the pathogenesis of CVD or if other mediators are involved. Understanding the causal pathways between RA and CVD could have the potential to find treatment strategies for both conditions and improve the cardiovascular outcomes of RA patients [11, 13].

In light of the increasing interest in the connection between RA and CVD, it is imperative to conduct a systematic review to consolidate the findings of MR studies and offer a representation of the current knowledge landscape. This review aims not only to deepen the comprehension of the causal relationship between RA and CVD but also to direct future research.

## Methods

**Fig. 1:**
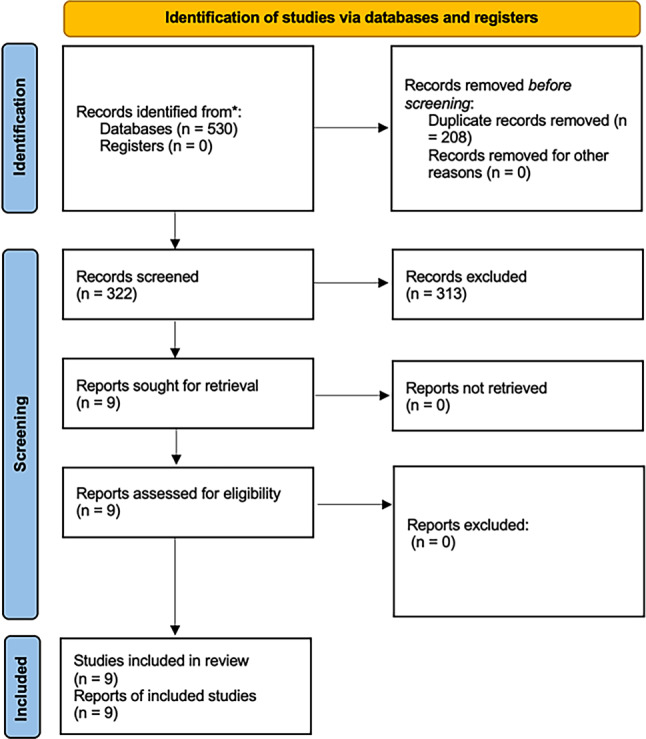
The PRISMA (Preferred Reporting Items for Systematic Reviews and Meta-Analyses) serves as a visual guide to transparently depict the selection process for the studies in the systematic review

The systematic review was registered on Open Science Framework (OSF). A comprehensive literature search was conducted using PubMed, Cochrane and Web of Science databases. The search was conducted using the following search terms: “rheumatoid arthritis” AND “Mendelian.” Two reviewers (MA and BW) independently assessed the eligibility of these studies based on predefined inclusion and exclusion criteria. The search was completed as of 15 October 2023. All studies conducted up to that date were considered for the review. The inclusion criteria focused on studies investigating the association between RA and CVD using MR methods. Studies were excluded if they did not meet these criteria, if they were reviews or comments, if they were not available in full text, or if they were not fully published in English. Additionally, the MR studies had to specifically address cardiovascular risk or diseases. The two reviewers identified the studies that met the inclusion criteria and were deemed suitable for the systematic review based on relevance, study design and the use of MR to examine the relationship between RA and CVD. The search process is depicted in the Prisma flow chart (Fig. 1). The selected studies are shown here:

1. Guo HY, Wang W, Peng H, Yuan H. Bidirectional two-sample Mendelian randomization study of causality between rheumatoid arthritis and myocardial infarction. Frontiers in immunology. 2022;13:1017444 [14].
2. Jia Y, Zhang K, Shi M, Guo D, Yang P, Bu X, et al. Associations of Rheumatoid Factor, Rheumatoid Arthritis, and Interleukin‑6 Inhibitor with the Prognosis of Ischemic Stroke: a Prospective Multicenter Cohort Study and Mendelian Randomization Analysis. Translational Stroke Research. 2023 [15].
3. Lin X, Zhou M, Zhang C, Li J. Genetically Determined Rheumatoid Arthritis May Not Affect Heart Failure: Insights from Mendelian Randomization Study. Global Heart. 2023;18(1):43 [16].
4. Nie Q, Luo Q, Yan W, Zhang T, Wang H, Wu J. Rheumatoid arthritis and coronary atherosclerosis: a two-sample Mendelian randomization study. Frontiers In Cardiovascular Medicine. 2023;10 [17].
5. Study Qiu S, Li M, Jin S, Lu H, Hu Y. Rheumatoid Arthritis and Cardio-Cerebrovascular Disease: A Mendelian Randomization Study. Frontiers in Genetics. 2021;12:745224 [18].
6. Wang M, Chao C, Mei K, Di D, Qian Y, Wang B, et al. Relationship between rheumatoid arthritis and cardiovascular comorbidity, causation or co-occurrence: A Mendelian randomization study. Frontiers In Cardiovascular Medicine. 2023;10 [19].
7. Wang M, Mei K, Chao C, Di D, Qian Y, Wang B, et al. Rheumatoid arthritis increases the risk of heart failure-current evidence from genome-wide association studies. Frontiers in Endocrinology. 2023;14:1154271 [20].
8. Yuan S, Carter P, Mason AM, Yang F, Burgess S, Larsson SC. Genetic Liability to Rheumatoid Arthritis in Relation to Coronary Artery Disease and Stroke Risk. Arthritis & Rheumatology (Hoboken, NJ). 2022;74(10):1638–47 [21].
9. Zhang K, Jia Y, Wang R, Guo D, Yang P, Sun L, et al. Rheumatoid arthritis and the risk of major cardiometabolic diseases: a Mendelian randomization study. Scandinavian Journal of Rheumatology. 2023;52(4):335–41 [22].

To evaluate the quality of the selected studies an adapted critical appraisal checklist for MR studies in CVD and RA research. The original checklist was introduced in the paper “Reading Mendelian randomization studies: a guide, glossary, and checklist for clinicians” [11]. This checklist was partly tailored to assess the methodology, outcomes and implications of each MR study within the framework of RA as a potential risk factor for CVD. The authors first ensured that the checklist questions directly pertained to the central research topic, which focused on the connection between RA and CVD. Next, adjustments were made to the questions to enhance the specificity, aligning them with the study’s objectives. To facilitate organization and accessibility, the questions were grouped into categories with appropriate headings, following the structure of the original tool. For clarity and ease of reference, numerical identifiers were also assigned to each question, akin to the format used in the original checklist. Finally, the adapted questions were presented in a format that enables a straightforward evaluation, with response options of “yes,” “no,” or “not available”. The checklist included 19 questions pertaining to core MR assumptions, methods reporting, data presentation, interpretation and clinical implications (Adapted Critical Appraisal Tool—Supplementary Material).

The categorization of evidence robustness is based on a tool developed by Markozannes et al., who conducted a systematic review of MR studies on cancer risk. This categorization includes five predefined causality levels (robust, probable, suggestive, insufficient evidence or non-evaluable) [23]. It relies on data from both the primary MR analysis and at least one of the following methods: MR-Egger, weighted median (WM), MRPRESSO and Mendelian randomization with pleiotropy score. The analysis was done by two investigators (MA and BW). In studies 2 and 8, the results are considered robust when MR Egger is not included. Because it was found that MR Egger produced results that partially differed from other analytical methods, which can be attributed to differences in the underlying modelling techniques. In such cases, the robustness analyses both with and without MR Egger are provided.

## Results

This search yielded 530 potential studies. After removing duplicate entries, a total of 322 unique studies remained and 9 studies met the inclusion criteria and were thus analyzed in the systematic review. The search process and study selection are shown in a PRISMA flowchart (Fig. 1; [24]).

Out of the nine studies eight originated from Chinese research groups, with one coming from Sweden. The publications spanned from 2021 to 2023, with 6 studies conducted in the year 2023. The sample sizes (*n*) for the RA cases ranged from 12,838 to 462,933. The number of utilized single nucleotide polymorphisms (SNP) ranged from 8 to 142. All studies were conducted using European ancestry data and were using two-sample Mendelian randomization (TSMR). The analysis of the conducted sensitivity analyses showed that studies 4 and 5 demonstrated robust results, meeting the criteria of both statistical significance and directional consistency. Initially, studies 2 and 8 were categorized as probable but when MR Egger was not included, they also exhibited robustness; however, study 3 was rated as insufficient due to the absence of statistical significance in the findings. Despite variations in statistical significance across different methods, the remaining studies were classified as probable because they consistently showed directional consistency. Notably, none of the studies fell into the not evaluable category as all of them underwent a rigorous sensitivity analysis to ensure comprehensive assessment (Table 1).

**Table 1:** The robustness analysis focused on evaluating whether all methods yielded consistent and statistically significant results across the studies

	Not evaluable	Insufficient	Suggestive	Probable	Robust
Study 1	–	–	–	Probable	–
Study 2	–	–	–	Probable (with MR Egger)	Robust (without MR Egger)
Study 3	–	Insufficient	–	–	–
Study 4	–	–	–	–	Robust
Study 5	–	–	–	–	Robust
Study 6	–	–	–	Probable	–
Study 7	–	–	–	Probable	–
Study 8	–	–	–	Prob. (with MR Egger)	Robust (without MR Egger)
Study 9	–	–	–	Probable	–

In our adapted questionnaire, question 1 was only answered with “yes” in studies where outcomes were subsequently assessed as robust. This applies to studies 2, 4, 5, and 8. In study 4, the answer “no” was given for question 5, as this was interpreted to suggest the utilization of two distinct sample populations. This interpretation was based on the study’s description, which indicated the selection of genetic variants associated with RA from GWAS. Subsequently, genetic data for coronary atherosclerosis were obtained from the UK Biobank. In the questionnaire, the following question could not be sufficiently addressed in any of the studies: “Will interventions at a specific age yield effect of the same magnitude?” This inadequacy stemmed from either the absence of analysis or a lack of comprehensive discussion on this particular aspect within the studies. Question 9 was only answered with “no” in study 5 and question 14 was only answered with “no” in study 2 because the study did not adequately address the possibility of weak instrument bias or confounding through horizontal pleiotropy. In our opinion the study did not thoroughly explore or account for these potential sources of bias. These results are shown in Table 2. Comprehensive information, including the sizes of the case and SNP samples, specifications regarding the dataset employed as well as the crucial statistics such as odds ratios (OR), confidence intervals (CI), and *p*-values, can be readily accessed in Tables 3, 4 and 5. Tables 2, 3, 4 and 5 are providing a detailed overview of the essential data points, facilitating a more thorough understanding of the study’s findings and methodology. Table 3 includes information about the primary authors, the journal names and the publication years.

**Table 2:** The responses were based on the critical appraisal questionnaire, with answers categorized as either “yes”, “no”, or “not available (–)”

	Study 1	Study 2	Study 3	Study 4	Study 5	Study 6	Study 7	Study 8	Study 9
Question 1	No	Yes	No	Yes	Yes	No	No	Yes	No
Question 2	Yes	Yes	Yes	Yes	Yes	Yes	Yes	Yes	Yes
Question 3	Yes	Yes	Yes	Yes	Yes	Yes	Yes	Yes	Yes
Question 4	Yes	Yes	Yes	Yes	Yes	Yes	Yes	Yes	Yes
Question 5	Yes	Yes	Yes	No	Yes	Yes	Yes	Yes	Yes
Question 6	Yes	Yes	Yes	Yes	Yes	Yes	Yes	Yes	Yes
Question 7	Yes	Yes	Yes	Yes	Yes	Yes	Yes	Yes	Yes
Question 8	Yes	Yes	Yes	Yes	Yes	Yes	Yes	Yes	Yes
Question 9	Yes	Yes	Yes	Yes	No	Yes	Yes	Yes	Yes
Question 10	Yes	Yes	Yes	Yes	Yes	Yes	Yes	Yes	Yes
Question 11	Yes	Yes	Yes	Yes	Yes	Yes	Yes	Yes	Yes
Question 12	Yes	Yes	Yes	Yes	–	Yes	Yes	Yes	Yes
Question 13	Yes	Yes	–	Yes	Yes	Yes	Yes	Yes	Yes
Question 14	Yes	No	–	Yes	Yes	–	Yes	Yes	Yes
Question 15	–	–	–	–	Yes	–	–	–	–
Question 16	No	No	No	–	–	No	No	No	–
Question 17	Yes	Yes	Yes	Yes	Yes	Yes	Yes	Yes	Yes
Question 18	Yes	Yes	–	Yes	Yes	Yes	–	Yes	Yes
Question 19	Yes	Yes	Yes	Yes	Yes	Yes	Yes	Yes	Yes

**Table 3:** The studies are described based on the published journal, the first author, the country of origin of the research group and the publication year. Subsequently, the main outcome was described and if associations were present, they were marked with “yes”

	Journal	First Author	Country	Year	Outcome	Associations
Study 1 [14]	Frontiers in Immunology	Hao-Yang Guo	China	2022	Myocardial infarction	Yes
Study 2 [15]	Translation Stroke Research	Yiming Jia	China	2023	Ischemic Stroke	Yes
Study 3 [16]	Global Heart Journal	Xueqi Lin	China	2023	Heart Failure	No
Study 4 [17]	Frontiers in Cardiovascular Medicine	Qiong Nie	China	2023	Coronary atherosclerosis	Yes
Study 5 [18]	Frontiers in Genetics	Shizheng Qiu	China	2021	Coronary Heart Disease (1) and Cardio-Cerebrovascular Disease	Yes
Study 6 [19]	Frontiers in Cardiovascular Medicine	Min Wang	China	2023	Ischemic Heart Disease (1) and Cardiovascular Comorbidity	Yes
Study 7 [20]	Frontiers in Endocrinology	Min Wang	China	2023	Heart Failure	Yes
Study 8 [21]	Arthritis and Rheumatology	Shuai Yuan	Sweden	2022	Coronary Artery Disease (1) and Stroke	Yes
Study 9 [22]	Scandinavian Journal of Rheumatology	Kaixin Zhang	China	2023	Coronary Artery Disease (1) and Cardiometabolic Disease	Yes

(1) If there were multiple outcomes the most relevant one for the topic was defined and marked with parentheses 1

**Table 4:** The analysis methods of Mendelian Randomization, the datasets used and the traceable ancestry are provided in the table

	Specified methods	Datasets	Ancestry
Study 1	TSMR/IVW/MR-Egger/simple mode/weighted mode	IEU OpenGWAS project	European
Study 2	TSMR/IVW/MR-PRESSO/MR-Egger/MR-Raps/maximum likelihood	GWAS Euro	European
Study 3	TSMR/IVW/MR-Egger/MR-PRESSO/leave one out	GWAS Euro (meta-analysis)	European
Study 4	TSMR/IVW/MR-Egger/maximum likelihood/multivariate MR/MR-PRESSO/leave-one-out	GWAS (Euro. RA/UK Biobank Atherios.)	European
Study 5	TSMR/IVW/MR-Egger/simple mode/leave-one-out	GWAS UK Biobank	European
Study 6	TSMR/IVW/MR-PRESSO/MR-Egger/MR-Raps/simple mode/weighted mode	GWAS Euro	European
Study 7	TSMR/IVW/MR-PRESSO/MR-Raps/maximum likelihood/MR-Egger regression	GWAS Euro	European
Study 8	TSMR/IVW/weighted median/MR-PRESSO/MR-Egger regression	International consortia, the UK Biobank, and the FinnGen consortium	European
Study 9	TSMR/IVW/MR-Egger/MR-PRESSO/simple median/weighted median	GWAS Euro./CARDIoGRAMplusC4D/MEGASTROKE	European

**Table 5:** The sample volume, the number of used SNPs, the odds ratios and *p*-values are provided. If multiple outcomes were analyzed, they are listed

	*n* RA cases	*n* RA Control	*n* Exposure (1) cases	*n* Exposure (1) Control	SNPs	OR + CI (IVW)	*p*-value (IVW)
Study 1	13,838	33,742	14,825	44,000	15	OR = 1.041; 95% CI = 1.007–1.076	*p* = 0.017
Study 2	n. a.	n. a.	n. a.	n. a.	142	OR = 1.090; 95% CI = 1.01–1.18	*p* = 0.021
Study 3	95,524	1,270,968	461,880	447,052	46	OR = 1.00; 95% CI = 0.99–1.02	*p* = 0.68
Study 4	14,361	43,923	14,334	346,860	54	OR = 1.0021; 95% CI = 1.0011–1.0031	*p* < 0.05
Study 5	462,933	457,732	10,693	451,187	8	OR (CAD) = 1.19; 95% CI = 1.01–1.39	*p* = 0.003
Study 6	14,361	43,923	12,801	187,840	82	OR (MI) = 1.0663; 95% CI = 1.022345–1.1122	*p* (MI) = 0.003724
Study 7	14,361	43,923	47,309	930,014	112	OR = 1.0226; 95% CI = 1.005495–1.039304	*p* = 0.009067
Study 8	14,361	43,923	122,733	424,528	70	OR (CAD) = 1.05; 95% CI = 1.02–1.08	*p* (CAD) = 0.001
Study 9	14,361	43,923	60,801	123,504	62	OR (CAD) = 1.02; 95% CI = 1.00–1.03	*p* (CAD) = 0.012

(1) If there were multiple outcomes the most relevant one for the topic was defined and marked with parentheses 1

*MI* myocardial infarction

Overall, the included studies illustrate that genetically determined RA is causally associated with an increased risk of CVD, including myocardial infarction (MI), ischemic heart disease (IHD), and CAD. Furthermore, associations between the occurrence of RA and T2D (Study 9) and hypertension (Study 5) were also identified. The consistent results across various MR methods and sensitivity analyses support the credibility of these causal relationships. Specifically, it was found that RA is causally linked to CAD. Additionally, the studies emphasized the importance of considering systemic inflammation as a potential mechanistic link between RA and these diseases. On the other hand, no significant causal relationship between RA and certain cardiovascular outcomes, such as ischemic stroke, atrial fibrillation (AF), or arrhythmias, was observed (study 6). These results suggest that the genetic factors contributing to RA are not directly associated with rhythmological conditions. Regarding the association between heart failure and RA, there were varying results. Study 3 did not find sufficient evidence to support a causal relationship between genetically predicted RA and heart failure; however, study 7 indicated an association.

## Discussion

This systematic review examines the relationship between RA and CVD through the lens of MR studies. By analyzing nine selected MR studies, the study aimed to provide a comprehensive understanding of this complicated interaction, highlighting the genetic point of view. The findings consistently indicate a causal association between genetically determined RA and an increased risk of cardiovascular outcomes. This highlights the necessity of recognizing RA as a significant risk factor for CVD and underscores the importance of proactive cardiovascular risk management in RA patients. Furthermore, the review explores the genetic connections between RA and other cardiovascular risk factors such as type 2 diabetes (T2D) and hypertension, underscoring the systemic impact of RA beyond joint-related symptoms. A notable revelation from the reviewed studies is the potential mechanistic role of systemic inflammation, driven by genetic factors in mediating the RA-CVD relationship. Chronic inflammation is progressively seen as a substance in the development of atherosclerosis, suggesting that targeting inflammation might be a promising strategy to relieve cardiovascular risk in RA patients. The genetic mechanisms in RA should be more investigated in rheumatology to enhance understanding of this aspect of the disease’s development. Further research in this area is essential for advancing personalized treatment strategies and improving patient outcomes. The association between RA and CVD is likely more pronounced due to the interplay between inflammation and atherosclerosis. This interaction is not observed in the case of heart failure, suggesting different underlying mechanisms [25, 26].

For sure it is important to recognize variations in study results which highlight the need for further research to systematically clarify these genetic causal relationships. Potential mechanisms beyond chronic inflammation must be considered, including the role of the different RA treatments in modulating CVD risk.

Limitations of the reviewed studies include population specificity, potential pleiotropy and the need for validation in diverse populations, highlighting the importance of cautious interpretation and further investigation to confirm and expand upon the results. It must be mentioned that all genetic analyses were from European ancestries and that gender differences were not analyzed. The strengths of the study lie in its thorough examination of all MR studies from recent years and the study demonstrates a current and informed overview of the field. The analyzed studies have used an extensive dataset, which offers a substantial pool of data for analysis. Additionally, the study sheds light on a genetic connection that is currently not well understood. A risk of bias module was tailored specifically for the MR studies to analyze the risk of reverse causality offering robust evidence.

In summary, this systematic review of Mendelian randomization studies provides valuable insights into the complex interplay between rheumatoid arthritis and cardiovascular diseases focusing on the genetic aspects. The consistent findings across the selected MR studies reinforce the association between RA and elevated cardiovascular risk, particularly CAD. Despite limitations the studies underscore the necessity of proactive cardiovascular risk management in RA patients and the potential benefits of interventions targeting inflammation.

## Supplementary Information

The supplementary information encompasses the adapted critical appraisal tool, complete with answered questions. This tool has been tailored to fit the specific context of our study, providing thorough evaluations and insights

## Data Availability

The authors are willing to provide data for specific reasonable requests from colleagues upon prior consultation.
