# Supplementary material for: Genetic relationship between rheumatoid arthritis and cardiovascular diseases: A systematic review of Mendelian randomization studies
Source: Wien Klin Wochenschr. 2024 Jul 26;137(9-10):272–8. doi: 10.1007/s00508-024-02392-8 (PMC12081514; doi:10.1007/s00508-024-02392-8)
Supplement: Supplementary file 1 — The supplementary information encompasses the adapted critical appraisal tool, complete with answered questions. This tool has been tailored to fit the specific context of our study, providing thorough evaluations and insights [file 508_2024_2392_MOESM1_ESM.docx]

Supplementary material

**Adapted Critical Appraisal Tool**

1. Is there sufficient evidence that the genetic variants used in the study are robustly associated with rheumatoid arthritis as a risk factor for cardiovascular diseases?

[Yes / No / Not Available]

1. Did the study assess whether the genetic variants used are also associated with potential confounders relevant to cardiovascular diseases and if so, did they present this relationship?

[Yes / No / Not Available]

1. Did the study consider the possibility of genetic variants affecting the outcome through alternative pathways (horizontal pleiotropy) and did they employ alternative Mendelian randomization approaches or use of "negative control" populations to investigate this further?

[Yes / No / Not Available]

1. Were the effect alleles and other alleles consistently coded in the same direction for the exposure (rheumatoid arthritis) and outcome (cardiovascular diseases) in the study?

[Yes / No / Not Available]

1. Were the two samples drawn from the same population?

[Yes / No / Not Available]

1. Were the two samples independent?

[Yes / No / Not Available]

1. Was the analysis restricted to independent variants?

[Yes / No / Not Available]

1. Did the authors present the results as a genetic association?

[Yes / No / Not Available]

1. Did they conduct a comparison between the instrumental variable estimate and the conventional observational estimate, if they provided both? [Yes / No / Not Available]
2. Did the authors provide sensitivity analyses?

[Yes / No / Not Available]

1. Did the authors manually pick and choose which SNPs go into the instrument to tackle pleiotropy?

[Yes / No / Not Available]

1. If so, is the approach and justification clear?

[Yes / No / Not Available]

1. Did the authors provide the data that they used (especially for Mendelian randomization analyses conducted at the summary level) in a supplement to allow researchers to reproduce their findings?

[Yes / No / Not Available]

1. If the Mendelian randomization estimate suggests that rheumatoid arthritis is a risk factor for cardiovascular diseases, did the study consider the possibility of weak instrument bias or confounding through horizontal pleiotropy as potential sources of the association? [Yes / No / Not Available]
2. If the Mendelian randomization estimate differs from the observational estimate and provides limited evidence of rheumatoid arthritis as a risk factor for cardiovascular diseases, did the study explore whether this discrepancy could be attributed to weak instrument bias, different sample characteristics, or negative confounding due to pleiotropy? [Yes / No / Not Available]
3. Mendelian randomization provides estimates of the effects of the risk factor over a lifetime and the numerical effect estimates may not be clinically meaningful. Will interventions at a specific age have the same sized effects?

[Yes / No / Not Available]

1. Are the 95% confidence intervals of the Mendelian randomization estimate sufficiently precise to identify the observational estimate and a clinically meaningful difference? [Yes / No / Not Available]
2. Do the results triangulate with other forms of evidence? [Yes / No / Not Available]
3. Could a clinical trial be conducted to provide definitive evidence for the treatment of rheumatoid arthritis and its potential impact on cardiovascular health? [Yes / No / Not Available]
